# Supplementary material for: Mechanosensor-mediated Hsp70 phosphorylation orchestrates the landscape of the heat shock response
Source: Nat Commun. 2025 Dec 13;17:507. doi: 10.1038/s41467-025-67204-7 (PMC12804701; doi:10.1038/s41467-025-67204-7)
Supplement: Supplementary file 1 — Supplementary Information [file 41467_2025_67204_MOESM1_ESM.pdf]

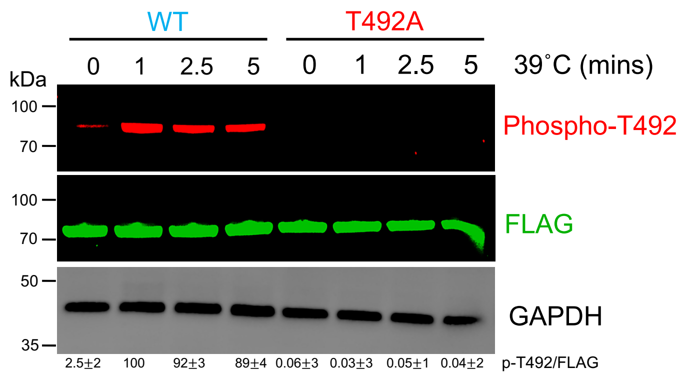

Supplementary Figure 1. Phosphorylation of T492 occurs rapidly in response to heat shock. Lysate from FLAG-WT Ssa1 cells (GPD promoter) or FLAG-T492A (GPD promoter) Ssa1 cells treated at 39 ˚C for the indicated times was analyzed by Western Blotting using antisera to either FLAG or phospho-T492. Source data are provided as a Source Data file.

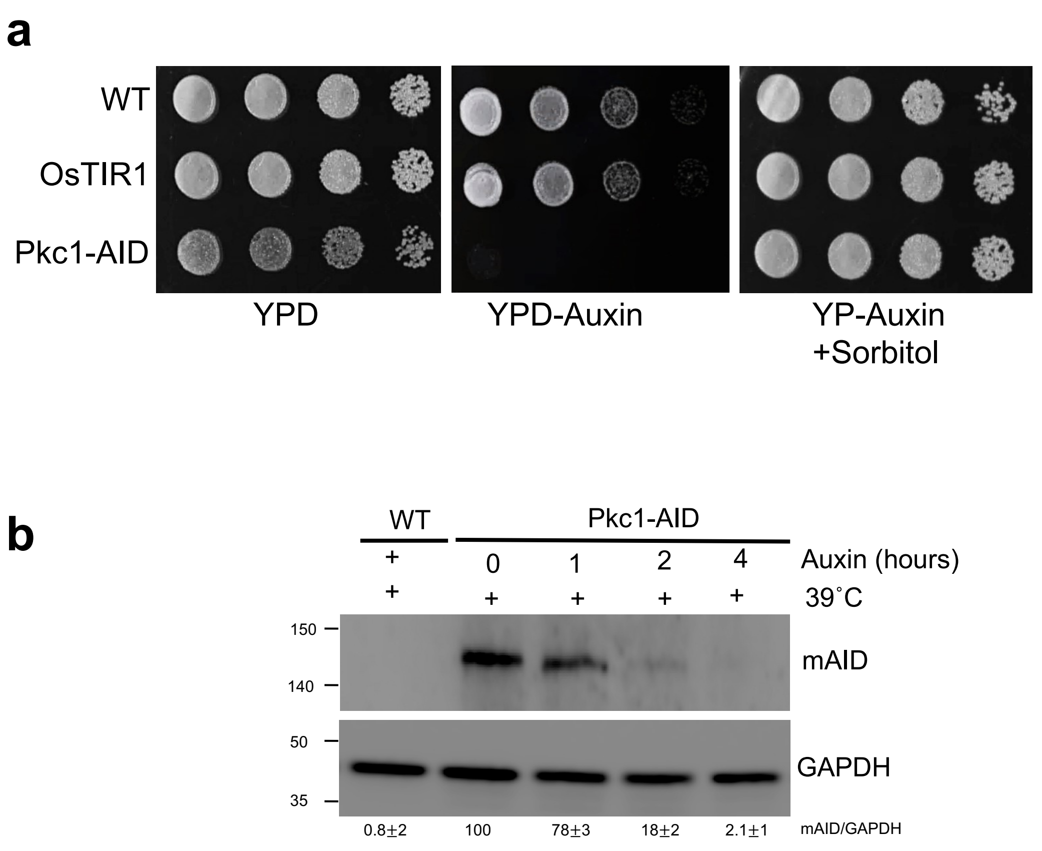

Supplementary Figure 2. Validation of the Pkc1-AID strain. a, The Pkc1-AID strain phenocopies loss of Pkc1upon addition of auxin. The indicated cells were grown to mid-log phase and then tenfold serially diluted onto either YPD, YPD+Auxin or YPD+Auxin+1M sorbitol. The plates were incubated for 3 days and then photographed. **b**, Pkc1-AID is degraded in response to auxin. Cells were grown as in Figure 2d and then protein was extracted via bead beating. Lysates were analyzed by Western Blotting usingantisera to either AID or GAPDH. Source data are provided as a Source Data file.

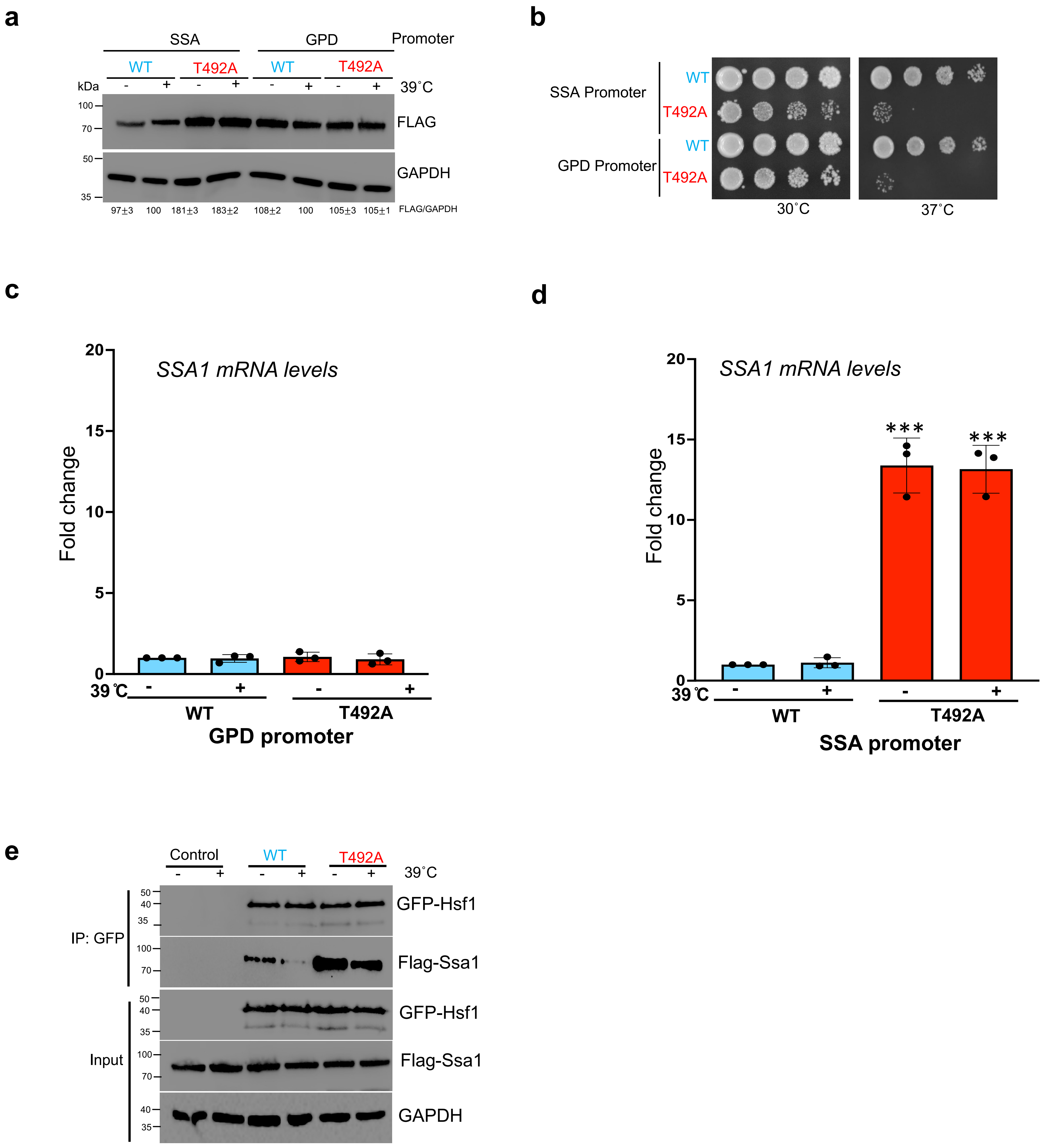

Supplementary Figure 3. Mutation of T492 impacts the heat shock response. **a**, The increased abundance of T492A Ssa1compared to WT can be prevented by using a constitutive promoter. Lysate from cells expressing either FLAG-WT or T492A (GPD promoter) or FLAG-WT or T492A (SSA promoter) left untreated at 25 ˚C or heat shocked at39 ˚C for 30 minutes were analyzed by Western Blotting using antisera to either FLAG or GAPDH. **b**, Cells expressing the Ssa1 T492A mutation are sensitive to elevated temperatures when expressed from either SSA orGPD promoter. The indicated cells were grown to mid-log phase and then tenfold serially diluted onto YPD and incubated at either 25 ˚C or 37 ˚C. The plates were incubated for 3 days and then photographed. **c**, The transcription of WT and T492A *SSA1* is equal when driven by the GPD promoter. Yeast strains expressing FLAG-Ssa1 and FLAG-T492A from constitutive GPD promoter were grown overnight, heat shocked at 39°C for 30 minutes, and RNA extracted using GeneJet kit. cDNA was synthesized from 1 µg RNA using iScript reversetranscriptase, then analyzed by qPCR on ABI Fast 2000 using SYBR Green. *SSA1* signals were normalized to *ACT1* with WT set as onefold. Data represent mean ± standard deviation from n=3 samples. Statistical significance was determined by two-way ANOVA with multiple comparisons test. ***p ≤ 0.001 **d**, T492A *SSA1* transcription is elevated compared to *SSA1* when driven by the native SSA promoter. Yeast strains expressing FLAG-Ssa1 and FLAG-T492A from native SSA promoter were grown overnight, heat shocked at 39°C for 30minutes, and RNA extracted using GeneJet kit. cDNA was synthesized from 1 µg RNA using iScript reverse transcriptase, then analyzed by qPCR on ABI Fast 2000 using SYBR Green. *SSA1* signals were normalized to *ACT1* with WT set as onefold. Data represent mean ± SD from three replicates. **e**, T492 phosphorylation mediates the Ssa1-Hsf1 interaction. GFP-Hsf1 was purified from FLAG-WT or T492A yeast (GPD promoter) under either untreated or heat-shocked conditions using GFP-Trap beads. Lysates and immunoprecipitates wereanalyzed by Western Blot using antisera to GFP or FLAG epitopes and GAPDH as loading control. Source data are provided as a Source Data file.

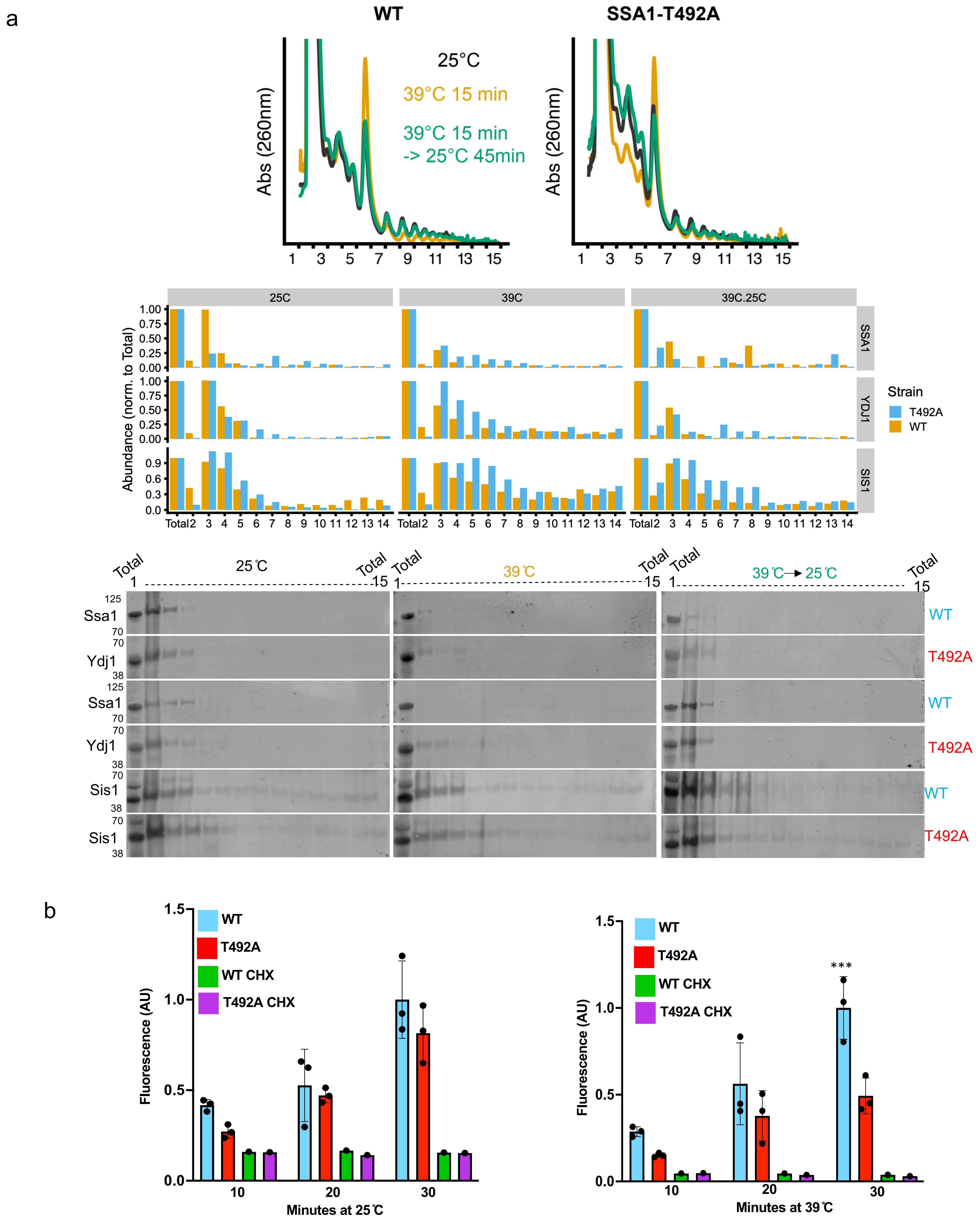

Supplementary Figure 4. T492 phosphorylation impacts heat-induced translation in GPD-driv en FLAG-Ssa1strains. a, Interaction of Ssa1, Ydj1 and Sis1 with polysome fractions in untreated, heat-shocked and recovery conditions. **b**, Translation was measured by incubating cells with the methionine analog homopropargylglycine (HPG) for the indicated time and then fluorescently labeling the cells with click chemistry. Control cells were incubated with the translation inhibitor cycloheximide (CHX). The changes in the fluorescence at the indicated time and temperature were statistically tested for significance using two-way ANOVA (***p ≤ 0.001). Source data are provided as a Source Data file.
