# Supplementary material for: Mechanosensor-mediated Hsp70 phosphorylation orchestrates the landscape of the heat shock response
Source: Nat Commun. 2025 Dec 13;17:507. doi: 10.1038/s41467-025-67204-7 (PMC12804701; doi:10.1038/s41467-025-67204-7)
Supplement: Supplementary file 2 — Description of Additional Supplementary Files [file 41467_2025_67204_MOESM2_ESM.pdf]

A Description of Additional Supplementary Files

File Name: Supplementary Data 1

Description: Yeast strains used in this study

File Name: Supplementary Data 2

Description: Plasmids used in this study

File Name: Supplementary Data 3

Description: Ssa1 interactors determined by Mass Spectrometry

File Name: Supplementary Data 4

Description: Primers for RT-PCR

File Name: Supplementary Data 5

Description: Full plasmid sequences for constructs made during the course of this study
