# Supplementary material for: Mechanosensor-mediated Hsp70 phosphorylation orchestrates the landscape of the heat shock response
Source: Nat Commun. 2025 Dec 13;17:507. doi: 10.1038/s41467-025-67204-7 (PMC12804701; doi:10.1038/s41467-025-67204-7)
Supplement: Supplementary file 8 — Reporting Summary [file 41467_2025_67204_MOESM8_ESM.pdf]

nature portfolio

Corresponding author(s): Andrew Truman

Last updated by author(s): 11/18/2025

Reporting Summary

Nature Portfolio wishes to improve the reproducibility of the work that we publish. This form provides structure for consistency and transparency in reporting. For further information on Nature Portfolio policies, see our Editorial Policies and the Editorial Policy Checklist.

Statistics

For all statistical analyses, confirm that the following items are present in the figure legend, table legend, main text, or Methods section.

n/a	Confirmed	
[ ]	[x]	The exact sample size (n) for each experimental group/condition, given as a discrete number and unit of measurement
[ ]	[x]	A statement on whether measurements were taken from distinct samples or whether the same sample was measured repeatedly
[x]	[ ]	The statistical test(s) used AND whether they are one- or two-sided<br>*Only common tests should be described solely by name; describe more complex techniques in the Methods section.*
[x]	[ ]	A description of all covariates tested
[x]	[ ]	A description of any assumptions or corrections, such as tests of normality and adjustment for multiple comparisons
[ ]	[x]	A full description of the statistical parameters including central tendency (e.g. means) or other basic estimates (e.g. regression coefficient) AND variation (e.g. standard deviation) or associated estimates of uncertainty (e.g. confidence intervals)
[ ]	[x]	For null hypothesis testing, the test statistic (e.g. F, t, r) with confidence intervals, effect sizes, degrees of freedom and P value noted<br>*Give P values as exact values whenever suitable.*
[x]	[ ]	For Bayesian analysis, information on the choice of priors and Markov chain Monte Carlo settings
[x]	[ ]	For hierarchical and complex designs, identification of the appropriate level for tests and full reporting of outcomes
[x]	[ ]	Estimates of effect sizes (e.g. Cohen's d, Pearson's r), indicating how they were calculated

Our web collection on statistics for biologists contains articles on many of the points above.

Software and code

Policy information about availability of computer code

Data collection	N/A
Data analysis	Data processing and analyses were performed using GraphPad Prism (version 7). Image analysis and preparation was done using Softworx 6.5 (Cytivia) and FIJI ImageJ

For manuscripts utilizing custom algorithms or software that are central to the research but not yet described in published literature, software must be made available to editors and reviewers. We strongly encourage code deposition in a community repository (e.g. GitHub). See the Nature Portfolio guidelines for submitting code & software for further information.

Data

Policy information about availability of data

All manuscripts must include a data availability statement. This statement should provide the following information, where applicable:

- Accession codes, unique identifiers, or web links for publicly available datasets
- A description of any restrictions on data availability
- For clinical datasets or third party data, please ensure that the statement adheres to our policy

Provide your data availability statement here.

Research involving human participants, their data, or biological material

Policy information about studies with human participants or human data. See also policy information about sex, gender (identity/presentation), and sexual orientation and race, ethnicity and racism.

Reporting on sex and gender	N/A
Reporting on race, ethnicity, or other socially relevant groupings	N/A
Population characteristics	N/A
Recruitment	N/A
Ethics oversight	N/A

Note that full information on the approval of the study protocol must also be provided in the manuscript.

Field-specific reporting

Please select the one below that is the best fit for your research. If you are not sure, read the appropriate sections before making your selection.

☒ Life sciences ☐ Behavioural & social sciences ☐ Ecological, evolutionary & environmental sciences

For a reference copy of the document with all sections, see nature.com/documents/nr-reporting-summary-flat.pdf

Life sciences study design

All studies must disclose on these points even when the disclosure is negative.

Sample size	N/A
Data exclusions	No data were excluded
Replication	All experiments were repeated at least in triplicate
Randomization	Not applicable for yeast experiments
Blinding	Not applicable for yeast work

Reporting for specific materials, systems and methods

We require information from authors about some types of materials, experimental systems and methods used in many studies. Here, indicate whether each material, system or method listed is relevant to your study. If you are not sure if a list item applies to your research, read the appropriate section before selecting a response.

Materials & experimental systems

n/a	Involved in the study	
☐	☒	Antibodies
☐	☒	Eukaryotic cell lines
☒	☐	Palaeontology and archaeology
☒	☐	Animals and other organisms
☒	☐	Clinical data
☒	☐	Dual use research of concern
☒	☐	Plants

Methods

n/a	Involved in the study	
☒	☐	ChIP-seq
☒	☐	Flow cytometry
☒	☐	MRI-based neuroimaging

Antibodies

Antibodies used	Anti- PhosphoT492 (21st-century biochemicals), Anti-PGK1 (Thermo #22C5D8), Anti FLAG (Sigma, #F1365), Anti-His (QIAGEN #34670), Anti-GAPDH (Thermo MA5-15738), Anti-Hsc70 (Rockland immunochemicals #200301F64), Anti-HA (Thermo #26183), Anti-Ydj1 (StressMarq #SMC-166D), Anti-Sis1(Gift from Dr. E Craig), Anti-Hsp26 (Gift from Dr. J Buchner), Anti-GFP (Roche #1814460), Anti-Mpk1 (SCBT #133189) and Anti-phospho Mpk1 (Cell-signaling #4695).
Validation	Each antibody we validated using a control without that tag or protein present. For the phospho-T492, we validate this using a T492 non-phosphorylatable negative control. All antibodies in this study were validated by the manufacturer prior to purchase and use.

Eukaryotic cell lines

Policy information about cell lines and Sex and Gender in Research

Cell line source(s)	MATa (MH272) ssa1Δ::trp1 ssa2::HisG ssa3::HisG ssa4::HisG (ssa1-4) [YCPlac33 SSA1] Fig.1c,d,f Fig. 2c Fig. 4c Fig.7a,d Jaiswal 2011<br>MATa EG123 ura3-52 leu2-3,112 trp1-1 his4 can1r Fig.2b,e Dr. David Levin lab<br>MATa EG123 hsc77Δ (wsc1)::LEU2 Fig.2b Dr. David Levin lab<br>MATa EG123 mid2Δ ::URA3 Fig.2b Dr. David Levin lab<br>MATa EG123 bck1Δ ::URA3 Fig.2e Dr. David Levin lab<br>MATa EG123 mkk1Δ ::LEU2 mkk2Δ ::URA3 Fig.2e Dr. David Levin lab<br>MATa EG123 mpk1Δ :: TRP1 Fig.2e Dr. David Levin lab<br>MATa (MH272) ssa1Δ::trp1 ssa2::HisG ssa3::HisG ssa4::HisG (ssa1-4) [YCPlac33 SSA1]::HSP12-GFP-HIS3MX Fig.4f This study<br>MATa (MH272) ssa1Δ::trp1 ssa2::HisG ssa3::HisGssa4::HisG (ssa1-4) [YCPlac33 SSA1]::MKK1-3HA-HIS3MX Fig.7e This study<br>MATa (MH272) ssa1Δ::trp1 ssa2::HisG ssa3::HisGssa4::HisG (ssa1-4) [YCPlac33 SSA1]::MKK2-3HA-HIS3MX Fig.7f This study<br>MATa (MH272) ssa1Δ::trp1 ssa2::HisG ssa3::HisG ssa4::HisG (ssa1-4) [YCPlac33 SSA1] Fig.2d This study
Authentication	Yeast strains used in the study were verified by both autotrophic-marker checking and sequencing. Strains from the Yeast Deletion Collection contain unique 20mer molecular barcodes that were verified via DNA sequencing. All strains were maintained as archived glycerol stocks maintained at -80̊ C, and cultures older than four weeks were discarded and replaced by fresh streaks from stock
Mycoplasma contamination	N/A
Commonly misidentified lines (See ICLAC register)	N/A

Plants

Seed stocks	N/A
Novel plant genotypes	N/A
Authentication	N/A
